# Role of Immune Checkpoint Inhibitor Therapy in Advanced *EGFR*-Mutant Non-Small Cell Lung Cancer

**DOI:** 10.3389/fonc.2021.751209

**Published:** 2021-11-16

**Authors:** Nathaniel Wiest, Umair Majeed, Karan Seegobin, Yujie Zhao, Yanyan Lou, Rami Manochakian

**Affiliations:** ^1^ Department of Internal Medicine, Mayo Clinic, Jacksonville, FL, United States; ^2^ Division of Hematology and Oncology, Department of Internal Medicine, Mayo Clinic, Jacksonville, FL, United States

**Keywords:** Non-small cell lung cancer, lung cancer, EGFR, tyrosine kinase inhibitor, immune-mediated adverse effects, immune checkpoint inhibitor (ICI), cancer immunotherapies

## Abstract

Over the last decade, the treatment of advanced non-small cell lung cancer (NSCLC) has undergone rapid changes with innovations in oncogene-directed therapy and immune checkpoint inhibitors. In patients with epidermal growth factor receptor (EGFR) gene mutant (*EGFR*m) NSCLC, newer-generation tyrosine kinase inhibitors (TKIs) are providing unparalleled survival benefit and tolerability. Unfortunately, most patients will experience disease progression and thus an urgent need exists for improved subsequent lines of therapies. The concurrent revolution in immune checkpoint inhibitor (ICI) therapy is providing novel treatment options with improved clinical outcomes in wild-type *EGFR* (*EGFR*wt) NSCLC; however, the application of ICI therapy to advanced *EGFR*m NSCLC patients is controversial. Early studies demonstrated the inferiority of ICI monotherapy to EGFR TKI therapy in the first line setting and inferiority to chemotherapy in the second line setting. Additionally, combination ICI and EGFR TKI therapies have demonstrated increased toxicities, and EGFR TKI therapy given after first-line ICI therapy has been correlated with severe adverse events. Nonetheless, combination therapies including dual-ICI blockade and ICI, chemotherapy, and angiogenesis inhibitor combinations are areas of active study with some intriguing signals in preliminary studies. Here, we review previous and ongoing clinical studies of ICI therapy in advanced *EGFR*m NSCLC. We discuss advances in understanding the differences in the tumor biology and tumor microenvironment (TME) of *EGFRm* NSCLC tumors that may lead to novel approaches to enhance ICI efficacy. It is our goal to equip the reader with a knowledge of current therapies, past and current clinical trials, and active avenues of research that provide the promise of novel approaches and improved outcomes for patients with advanced *EGFR*m NSCLC.

## 1 Introduction

Lung cancer remains the leading cause of cancer-related death in the United States with an estimated 235,760 new cases and 131,880 new deaths in 2021 (1). Non-small cell lung cancer (NSCLC) represents approximately 85% of all lung cancer cases in the United States (2), and includes three major histologic subtypes: adenocarcinoma (ADC), squamous cell carcinoma (SCC), and large cell carcinoma (LCC). More than three quarters of patients with NSCLC have advanced (stage III or IV) disease at time of diagnosis, where clinical outcomes and survival have remained suboptimal (3). Fortunately, systemic treatment options for NSCLC have recently made significant improvements with advancements in oncogene-directed and ICI therapies. Data from the Surveillance, Epidemiology, and End Results (SEER) database demonstrate an approximate two-fold increase in 5-year survival for patients with lung cancer from 1973 to 2010 from 10.7 to 19.8% (4), consistent with improved treatment options, and this trend in improved patient outcomes is expected to continue. Targeted therapies for NSCLC are constantly evolving and there is significant interest in the potential interplay between immunotherapy and targeted therapies.

Multiple targetable genetic alterations have been identified in patients with NSCLC affecting the *EGFR, KRAS*, *BRAF*, *PIK3CA, ALK*, *ROS1*, *NRAS*, and *MET* genes among others (5). Lung ADC harbors more recognized oncogene mutations that SCC or LCC (6, 7), with up to 64% of metastatic lung ADC cases carrying a recognized oncogene driver mutation (8). The frequency of driver mutations is increased in females, never-smokers, and East Asian populations (6). Many clinical trials are underway to expand the number of targeted therapies, therapy combinations, and clinical contexts in which targeted therapies can be offered in NSCLC (9).

*EGFR* mutations are among the most common NSCLC driver mutations. EGFR is a receptor tyrosine kinase (RTK) that activates Ras/MAPK and PI3K/Akt cell signaling pathways and leads to cell proliferation, metastasis, and resistance to cell death when dysregulated (10). *EGFR* mutations are found in 19-23% of lung ADC in the United States and up to 64-67% of lung ADC in other regions including South East Asia and Peru (11). *EGFR* mutations are less common in lung SCC with a frequency of 2-10% and have only rarely been reported in cases of LCL (12, 13). Almost 90% of *EGFR* mutations are either deletions in exon 19 (ex19del) or leucine to arginine substation in exon 21 (L858R), with less common mutations occurring in exons 18, 20, and elsewhere (14). These mutations structurally activate EGFR signaling *via* different mechanisms and, critically, increase the binding affinity of various EGFR TKIs that inhibit mutant EGFR and spare wild-type EGFR at therapeutic concentrations (15, 16).

First-generation EGFR TKIs including erlotinib and gefitinib were first approved the U.S. Food and Drug Administration (FDA) in 2013 for first line use in metastatic *EGFR* ex19dels or L858R mutant NSCLC in 2013 after multiple studies demonstrated improved clinical outcomes compared to platinum-based chemotherapy (17, 18). The second generation, irreversible EGFR TKI afatanib was approved for first-line use in July 2013 (19). Despite improvement in PFS with first- and second-generation EGFR TKIs, clinical trials failed to uniformly demonstrate improvements in overall survival (OS) due to the development of TKI resistance occurring typically 10-14 months after treatment initiation (20). The most common mechanism of acquired resistance is the EGFR T790M mutation that inhibits the binding of first- and second-generation EGFR TKIs (21), though other mechanisms of resistance have been described (21). Given the predominance of EGFR T790M as the escape strategy for TKI resistance, third-generation EGFR TKIs including osimertinib were developed that are effective against EGFR containing the T790M substitution (16). Osimertinib was initially approved in 2015 for second-line treatment of EGFR T790M-mutant NSCLC after progression on first-line EGFR-TKIs; however, this approval was expanded to first-line use after the landmark FLAURA study demonstrated significantly increased PFS with osimertinib versus standard EGFR-TKIs of 18.9 versus 10.2 months (HR for PFS 0.46, 95% CI 0.37-0.57, *p*<0.001) and improved OS of 38.6 versus 31.8 months (HR for death 0.80, 95% CI 0.64-1.00, *p* = 0.046) (22–24). Given the impressive performance of osimertinib in both first- and second-line contexts, as well as its favorable side effect profile, it is now the standard of care for EGFR targeted therapy in *EGFR*m NSCLC (25). Active research is defining which *EGFR* mutations respond best to different EGFR TKIs and fourth generation inhibitors have been described that are under active investigation (26).

In addition to driver-mutation targeted therapies, the discovery and utilization of immune checkpoint (ICP) inhibitors in NSCLC has provided new and hopeful treatment options for many patients (27).The ICP describes an immunomodulatory process that downregulates T-cell effector responses and is mediated in part by the B7 ligand binding to the cytotoxic T-lymphocyte-associated protein-4 (CTLA-4) receptor and the programmed cell death ligands 1 and 2 (PD-L1 and PD-L2) binding to the programmed cell death protein 1 (PD-1) (28). While the ICP is normally a tissue protective mechanism that prevents autoimmunity, ICP activation is a strategy that many cancer types including NSCLC utilize to impede effective anti-tumor T-cell responses (29). Monoclonal antibodies that bind to CTLA4, PD-1, or PD-L1, known as ICIs, reduce activation of the PD-1/PD-L1 axis to remove inhibitory signals of anti-tumor T-cell activation. ICP blockade enhances anti-tumor T-cell mediated immune responses, especially in immunogenic tumors that rely on ICP activation to escape immune destruction (30, 31). An active challenge is to identify patients who will respond to ICIs before prescribing therapy, as ICIs can cause potent and wide-ranging immune-mediated adverse events (irAEs) including rash, endocrine abnormalities, and interstitial pneumonitis among many others (32).

There are multiple ICIs currently approved by the FDA for the treatment of NSCLC (33). ICIs were first approved in the second-line setting for metastatic NSCLC (mNSCLC) in March 2015 after nivolumab demonstrated superiority to docetaxel for squamous mNSCLC that progressed on platinum therapy (34). Subsequently, both pembrolizumab and atezolizumab were approved in second-line contexts for mNSCLC by 2016, and since then ICI indications have expanded and are approved in different combinations and settings (35). Pembrolizumab, atezolizumab, and combination of ipilimumab + nivolumab have obtained approval in first-line contexts (36). As such, there now exists a potent repertoire of immunotherapy strategies for patients with advanced NSCLC, though the question of how to integrate ICI therapy with targeted therapies in oncogene-driven NSCLC is an active area of research.

The role of ICI therapy in *EGFR*m advanced NSCLC is the focus of this review. We have discussed the role of ICI therapy in NSCLC with *ALK*, *ROS1*, *BRAF*, *c*-*MET*, *RET*, and *NTRK* mutations is separate, companion review in this journal. Below, we detail past and current clinical trials evaluating ICI therapy in advanced *EGFR*m NSCLC. We highlight different treatment sequences and combinations as well as subgroups that experienced either improved outcomes or unexpected toxicities. While the role of ICI therapy in *EGFR*m NSCLC is controversial, there is intriguing and hopeful evidence that certain combinations may prove beneficial, and active research is elucidating properties of *EGFR*m tumor biology and TME composition that we anticipate will lead to novel therapies in the near future.

## 2 Clinical Trials of Immune Checkpoint Therapy in Advanced EGFR Mutant NSCLC

A number of clinical trials have been performed utilizing ICIs in advanced *EGFR*m NSCLC. Initial studies on ICI first-line therapy were overall disappointing and did not reach the efficacy of *EGFR* TKIs. However, second- and later-line ICI therapy has demonstrated promise in certain contexts, with select subgroups demonstrating improved response to ICI strategies. Active clinical studies are addressing important questions including combinations of dual ICIs and ICI + Vascular Endothelial Growth Factor (VEGF) inhibitors + chemotherapy in progressive *EGFR*m NSCLC that are of significant interest to researchers and clinicians in the field. Clinical trials are summarized in **Tables 1**, **2** and are described in detail below.

**Table 1:** First-line ICI Clinical Trials in *EGFR*m NSCLC.

Trial	Phase	Intervention	Outcome	Safety	Reference	
**ICI Monotherapy**					
CheckMate 012	1	Nivolumab	ORR: 14% for *EGFR*m *vs* 30% for *EGFR*wt PFS: 1.8 *vs* 6.6 mo	G3-4^#^: 17%, G5: 0%	(37)	
NCT02879994	2	Pembrolizumab	ORR: 0%*	TRAE: 46%, no G4-5 6/7 patients had a TRAE on second-line EGFR TKI, including one G5 pneumonitis	(38)	
**ICI + Chemotherapy**						
CheckMate 012	1	Nivolumab + PT-DC	ORR: 17% for *EGFR*m *vs* 47% for *EGFR*wt PFS: 4.8 *vs* 7.5 mo OS: 20.5 *vs* 24.5 mo	G3-4^#^: 50%, G5: 0%. Pneumonitis most common TRAE (7%)	(39)	
**ICI + EGFR TKI Therapy**						
NCT02088112	1	Gefitinib + durvalumab dose escalation	ORR: 63.3%-70% PFS: 10.1-12.0 mo	TRAE: 100%, 17/40 high-grade hepatic AEs	(40)	
KEYNOTE-021	3	Pembrolizumab (P) + erlotinib (E) or gefitinib (G)	ORR: 41.7% P+E, 14.3% P + G PFS: 19.5 mo P+E, 1.4 mo P + G	P+E: TRAE: 100%, G3: 33.3%, no G4-5 P+G: TRAE: 85.7%, G3-4: 71.4% hepatotoxic AEs	(41)	
NCT02013219	1b	Atezolizumab + erlotinib	ORR: 75% PFS: 11.3 mo	G3-4^#^: 39%	(42)	
**Dual ICI Therapy**						
CheckMate 012	1	Nivolumab + ipilimumab	ORR: 50%	TRAE^#^: 72-82%, G3-4: 33-37%, no G5	(43)	

Indicated categories of trials with respective trial parameters are given. *1/11 patients initially reported to respond but was found to be EGFRwt. ORR, overall response rate; PFS, progression-free survival; OS, overall survival; TRAE, Treatment related adverse event; G, grade of toxicity; PT-DC, platinum-doublet chemotherapy; ^#^TRAEs for entire study population and not selected for EGFRm patients.

**Table 2:** Second-line or later ICI Clinical Trials in *EGFR*m NSCLC.

Trial	Phase	Intervention	Outcome	Safety	Reference	
**ICI Monotherapy**					
KEYNOTE-010	3	Pembrolizumab (P) *vs* docetaxel (D)	HR for PFS^$^ with P *vs* D: 1.79 in *EGFR*m *vs* 0.83 in *EGFR*wt HR for OS with P *vs* D: 0.88 vs 0.66	P: G3-5^#^: 13-16% D: G3-5: 35%	(44)	
CheckMate 057	3	Nivolumab (N) *vs* docetaxel (D)	HR for OS^$^ with N *vs* D: 1.18 for *EGFR*m *vs* 0.66 in *EGFR*wt	N: G3-5^#^: 10% D: G3-5: 54%	(45)	
POPLAR	2	Atezolizumab (A) *vs* docetaxel (D)	HR for OS^$^ with A *vs* D: 0.99 for *EGFR*m *vs* 0.70 in *EGFR*wt	A: G3-4^#^: 40%, G5: 4% D: G3-4: 53%, G5: 4%	(46, 47)	
KEYNOTE-001	1b	Pembrolizumab	PFS^$^: 6.0 mo in *EGFR*m *vs* 12.1 mo in *EGFR*wt	N.R.**	(48)	
PACIFIC	3	Durvalumab	HR for PD^$^ or death: 0.76 in *EGFR*m *vs* 0.47 in *EGFR*wt	TRAE: 96.8%, G3-4: 29.9%	(49)	
ATLANTIC	2	Durvalumab	ORR for *EGFR*m/*ALK*m: 3.6% PD-L1 TPS <25%, 12.2% for PD-L1 ≥25% OS for *EGFR*m/*ALK*m: 9.9 mo PD-L1 TPS <25%, 13.3 mo for PD-L1 ≥25%	G3-4: 5%	(50)	
**ICI + Chemotherapy**						
NCT03513666	2	Toripalimab + PT-DC	ORR: 50% PFS: 7.0 mo	G3-5: 55%, including neutropenia (48%), leukopenia (20%), and anemia (13%)	(51)	
**ICI + EGFR TKI Therapy**						
CheckMate 012	3	Nivolumab + erlotinib	ORR: 15%^&^ PFS: 5.1 mo OS: 18.7 mo	G3: 24%, no G4-G5	(52)	
TATTON	1b	Durvalumab + osimertinib	ORR: 43%	TRAE: 100%, G3-5: 48%. ILD occurred in 22% with G≥3 ILD in 8.7%	(53)	
CAURAL	3	Durvalumab + osimertinib	ORR: 64%	TRAE: 100%, G3-5: 8%. One G2 ILD reported	(54)	
**Dual ICI Therapy**						
KEYNOTE-021	1/2	Pembrolizumab + ipilimumab	ORR: 10% for *EGFR*m *vs* 30% for *EGFR*wt	TRAE^#^: 98%, G3-G5: 49%, one G5 pancreatitis	(55)	
**ICI + VEGF Inhibitor + Chemotherapy**						
IMpower150	3	Atezolizumab (A) + bevacizumab (B) + carboplatin-paclitaxel (CP)	ORR^##^: 70.6% for ABCP, 35.6% for ACP, 41.9% for BCP OS: NR for ABCP^##^, 17.5 mo for BCP	G3-4: 64% of ABCP, 68% of ACP, and 64% of BCP	(56)	
NCT03647956	2	Atezolizumab + bevacizumab + pemetrexed-carboplatin	ORR: 62.5% PFS: 9.43 mo	G3-5: 37.5%, One G5 myocardial infarction, 7.5% blood clot	(57)	

Indicated categories of trials with respective trial parameters are given. ORR, overall response rate; TRAE, Treatment related adverse event; G, grade of toxicity. ^#^TRAEs for entire study population and not selected for EGFRm patients. ^$^ORR data not given for EGFRm subgroup. **Safety data were not reported in this long-term survival update report. PD, progressive disease; PT-DC, platinum-doublet chemotherapy. ^&^Authors note that these patients were all TKI treated for first-line. NR, not reached. ^##^These numbers refer to the subgroup of patients with sensitizing EGFR mutations.

### 2.1 First-Line ICI Therapy

Several small studies or subgroups of larger studies have evaluated first-line ICIs either alone or as combination therapy in advanced *EGFR*m NSCLC (**Table 1**). Overall, ICI first-line therapy is unhelpful compared to EGFR TKI monotherapy, especially given the outstanding safety profile of the third-generation EGFR TKI osimertinib for advanced *EGFR*m NSCLC patients with PFS reported at 18.9 months and OS reported at 38.6 months in the FLAURA trial (23). Additionally, combination ICI + EGFR TKI may have enhanced toxicity and ICI treatment before EGFR TKI administration may prime patients for significant later toxicities with second-line EGFR TKI treatment.

#### 2.1.1 ICI Monotherapy

Based on a subgroup of the KEYNOTE-001 trial, in which a small number of *EGFR*m, TKI-naïve patients experienced improved objective response rate (ORR) compared to TKI-pretreated patients, the follow up Phase II NCT02879994 trial evaluated pembrolizumab first-line therapy in TKI-naïve, *EGFR*m advanced NSCLC patients with PD-L1 positivity (TPS ≥ 1%) (38, 58). This trial was halted early due to futility, as only 1/11 initial patients experienced an OR and this patient was found on subsequent analysis to be *EGFR*wt. Importantly, 6/7 patients that switched to second line TKI therapy after PD on pembrolizumab experienced treatment-related adverse events (TRAEs) from the TKI (erlotinib), including one patient who experienced grade 5 pneumonitis. These results suggested potential toxicity of EGFR TKI therapy after pembrolizumab treatment in *EGFR*m NSCLC patients.

A small subgroup of patients in the CheckMate 012 study examining first-line nivolumab monotherapy were *EGFR*m (6/56, 11%). The ORR for *EGFR*m versus *EGFR*wt patients was 14% versus 30%, indicating a comparatively decreased efficacy of ICI therapy in the first-line for *EGFR*m patients (37).

#### 2.1.2 ICI + Chemotherapy

The CheckMate 012 trial compared niviolumab + platinum-doublet chemotherapy in *EGFR*wt versus *EGFR*m advanced NSCLC patients as first-line treatment (39). *EGFR*m patients experienced worse PFS (4.8 *vs* 7.5 months) and median OS (20.5 versus 24.5 months) compared to *EGFR*wt.

#### 2.1.3 ICI + EGFR TKI Therapy

The results of the phase 1 NCT02088112 study, a two-part, dose-escalation study with durvalumab + gefitinib as first-line therapy in advanced *EGFR*m NSCLC patients, were recently published (40). All patients in the study received gefitinib daily. In the dose escalation portion of the study (Part A), 3 patients were randomized to additionally receive durvalumab 3 mg/kg every 3 weeks and 13 were randomized to receive durvalumab 10mg/kg every 3 weeks. Grade 3/4 TRAEs were reported in 68.8% of dose-escalation patients, leading to discontinuation of combined treatment in 94% of patients in this phase. In the dose-expansion phase (Part B), 40 patients were recruited to one of two treatment strategies: 1) gefitinib + durvalumab 10 mg/kg every 2 weeks (Arm 1, 30 patients), or 2) gefitinib for 4 weeks followed by addition of durvalumab 10 mg/kg every 2 weeks (Arm 2, 10 patients). Median PFS was 10.1 months in Arm 1 (95% CI 5.5-15.2 months) and 12.0 months in Arm2 (95% CI: 2.7-15.6 months), which was not considered improved compared to gefitinib monotherapy studies. For example, the phase 4 update of the NCT01203917 first-line gefitinib study demonstrated PFS of 9.7-10.2 months with gefitinib monotherapy (59). 17/40 of the dose-expansion phase patients experienced high-grade hepatic events, suggesting an additive effect of gefitinib and durvalumab for hepatotoxicity. The authors noted a trend towards favorable PFS in patients with TPS ≥ 20% (HR 0.46, 95% CI 0.19-1.03), but overall concluded that their study did not support combination gefitinib + durvalumab as first line treatment in *EGFR*m advanced NSCLC.

The ongoing open-label, multicohort phase 1/2 KEYNOTE-021 study (NCT02039674) is evaluating pembrolizumab in combination with chemotherapy or immunotherapy in previously untreated stage IIIB/IV *EGFR*m NSCLC patients (41). Two cohorts were reported early and evaluated pembrolizumab + EGFR TKI: Cohort E included 12 patients treated with pembrolizumab + erlotinib and Cohort F included 7 patients treated with pembrolizumab + gefitinib. The pembrolizumab + gefitinib arm was permanently discontinued early due to safety concerns, as 5/7 patients (71.4%) had treatment-related elevations in ALT and AST. However, the pembrolizumab + erlotinib arm was found to be tolerated and this arm experienced an objective response rate (ORR) of 41.7%, which is similar to the ORR seen with erlotinib and pembrolizumab monotherapies. All (4/4) patients with TPS ≥ 50% had an objective response, whereas only 1/4 patients with TPS 1%-49% and 0/2 patients with TPS <1% responded to pembrolizumab + erlotinib (41).

The phase 1b NCT02013219 study evaluated first-line erlotinib + atezolizumab in TKI-naïve patients with *EGFR*m NSCLC (42). The preliminary report included 28 patients and demonstrated a median PFS of 11.3 months, which was similar to erlotinib monotherapy. However, 50% of patients experienced serious TRAEs including 39% of patients who experienced grade 3/4 events. These data suggest that combination atezolizumab + erlotinib enhances toxicity without significant additive benefit. We await the publication of full trial results make a more comprehensive assessment of this combination.

The Phase 1/2 CheckMate 370 study (NCT02574078) is evaluating nivolumab as maintenance or first-line + other standard of care therapies (60). Group D will compare erlotinib versus nivolumab + erlotinib. Results have not yet been announced.

#### 2.1.4 Dual ICI Therapy

As part of the multi-arm phase 1 CheckMate 012 (NCT01454102) trial, patients with chemotherapy-naïve Stage IIIB/IV NSCLC were randomized to receive different dose schedules of nivolumab and ipilimumab as first line treatment. 10-11% of patients in different arms had *EGFR* activating mutations. Of these, 50% (4/8) had objective responses with combined nivolumab + ipilimumab, including 3/3 (100%) of patients with TPS ≥ 50% (43). PFS data are not available. These results, while limited by small sample size, suggest that combination immune checkpoint inhibition therapy may more effectively sensitize *EGFR*-mutant, PD-L1-expressing NSCLC to immune-mediated destruction than ICI monotherapy.

### 2.2 Second-Line or Later ICI Therapy

Multiple clinical trials have been performed with single or dual agent ICI therapy after patients experienced progressive disease (PD) with EGFR TKIs (**Table 2**). Most of the earlier studies were single agent trials that did not demonstrate benefit versus chemotherapy. However, more recent trials have added intriguing combination ICI strategies that may yield enhanced benefit.

#### 2.2.1 ICI Monotherapy

In regard to single agent ICI as a second-line agent after PD on EGFR TKI, three studies explored different single agent ICIs versus docetaxel. KEYNOTE-010 included a small number of *EGFR*m advanced NSCLC patients (9%) who were randomized to receive 2mg/kg pembrolizumab, 10mg/kg pembrolizumab, or 75 mg/m^2^ docetaxel every three weeks after having progressed on at least two cycles of platinum-based chemotherapy and treatment with an EGFR TKI. The *EGFR*m patients experienced worse PFS with pembrolizumab versus docetaxel (HR for PFS 1.79, 95% CI 0.94-3.42, *p* not given), in contrast to the *EGFR*wt patients who had an improved pooled PFS (HR for PFS 0.83, 95% CI 0.71-0.98, *p* not given) (44). CheckMate 057 (NCT01673867) was a large phase 3 study of nonsquamous NSCLC patients who had progressed on or after platinum-doublet therapy that compared nivolumab 3mg/kg every two weeks versus docetaxel 75 mg/m^2^ every three weeks. *EGFR*m patients were allowed to have received previous treatment with an EGFR TKI. 14% of study participants were *EGFR*m positive and experienced worse OS with nivolumab compared to docetaxel (HR 1.18, 95% CI 0.69-2.00, *p* not given), whereas the *EGFR*wt patients experienced significant benefit to OS with nivolumab (HR 0.66, 95% CI 0.51-0.86, *p* not given) (45). The POPLAR phase 2 study compared atezolizumab versus docetaxel as second line therapy for NSCLC. Similar to the PD-1 inhibitor studies, atezolizumab improved OS compared to docetaxel among all NSCLC patients (HR 0.73, 95% CI 0.53-0.99, *p*=0.04) (46). However, as reported by Lee et al., the subgroup of *EGFR*m patients did not see an improvement in OS (HR for OS 0.70 in WT versus 0.99 in *EGFR*-mutant, compared to docetaxel) (47). Thus, PD-L1 inhibition, as with PD-1 inhibition, failed to demonstrate improvement in *EGFR*m patients in the second-line context, though ICI therapy was more well tolerated across all three studies with less TRAEs compared to docetaxel.

A meta-analysis published by Lee et al. combined the results of these three studies utilizing ICI single therapy versus docetaxel as second-line therapy. The *EGFR*m NSCLC patients overall did not benefit from ICI monotherapy compared to docetaxel, whereas *EGFR*wt patients experienced a significant benefit from ICI therapy (HR for OS 1.05 for *EGFR*m *vs* 0.66 for *EGFR*wt) (47), confirming that ICI monotherapy is not advantageous over chemotherapy in the second-line setting for *EGFR*m patients with PD on TKI/chemotherapy.

Hui et al. reported updated results from the KEYNOTE-001 study, which examined pembrolizumab efficacy across several settings for patients with NSCLC and PD-L1 TPS ≥ 1% (48). The subgroup of *EGFR*m patients who had previously received treatment had significantly less benefit from pembrolizumab than *EGFR*wt patients (median OS 12.1 months versus 6.0 months). PD-L1 overexpression (TPS ≥ 50%) did not rescue response to pembrolizumab in the *EGFR*m *vs EGFR*wt patients (median OS 6.5 *versus* 15.7 months) (48). These results suggested that PD-L1 is an imperfect biomarker to predict ICI response in previously treated *EGFR*m patients, as discussed in further detail below.

The PACIFIC trial (NCT02125461) was a phase 3 trial that assessed the addition of durvalumab consolidation therapy after definitive chemoradiotherapy (CRT) for patients with stage III NSCLC (49). *EGFR*m NSCLC patients did not have significant benefit from durvalumab consolidation therapy (HR for PD or death 0.76, 95% CI: 0.35-1.64) whereas the *EGFR*wt patients did experience benefit (HR for PD or death 0.47, 95% CI: 0.36-0.60). In the recently published four-year survival update of the PACIFIC trial, *EGFR*m NSCLC patients again did not demonstrate benefit from durvalumab consolidation (HR for PFS 0.84, 95% CI: 0.40-1.75) whereas the *EGFR*wt patients again demonstrated significant benefit (HR for PFS 0.51, 95% CI: 0.40-0.65) (61).

Similar to the PACIFIC trial, Aredo et al. recently published the results of a multi-center retrospective study of patients (n=13) with unresectable *EGFR*m NSCLC who received consolidation durvalumab after CRT (62). They compared these patients to a cohort of *EGFR*m NSCLC patients who instead received consolidation EGFR TKI after CRT (n=24). Median PFS was 10.3 months for the CRT + durvalumab cohort versus 26.1 months for the CRT + EKGFR TKI group (*p* = 0.023). Notably, six patients opted to switch to EGFR TKI after experiencing PD on CRT + durvalumab and one of these patients developed Grade 4 pneumonitis 17 days after initiating osimertinib, again highlighting the safety signal of initiating EGFR TKIs after ICI therapy.

The ATLANTIC trial was a phase 2 open-label trial of durvalumab monotherapy as third-line or later treatment in patients with advanced NSCLC (63). Enrolled patients had to have received at least two previous lines, with one platinum-containing regimen and a TKI if indicated. Cohort 1 included both *EGFR*m and *ALK*m NSCLC patients and was stratified by PD-L1 TPS: median OS was 13.3 months in the TPS ≥ 25% subcohort versus 9.9 months in the TPS < 25% subcohort. Notably, this was higher than in the *EGFR*wt and *ALK*wt Cohort 2 that demonstrated median OS of 10.9 versus 9.3 months for TPS ≥ 25% and TPS <25% subcohorts, respectively. The safety profile of *EGFR*m/*ALK*m cohort was similar to the *EGFR*wt/*ALK*wt profile, with 6-8% of patients experiencing Grade 3-4 TRAEs, supporting the safety of ICI administration after EGFR TKI treatment. Of note, the ATLANTIC trial has multiple limitations, including lack of descriptive statistics, single-arm design, and variations in testing platform for PD-L1 that made direct comparisons with other trials not possible (64). Despite this, the ATLANTIC trial suggested that ICI therapy may have efficacy in heavily pre-treated *EGFR*m NSCLC patients and further supported the safety of ICI therapy after patients progressed on TKI therapy.

#### 2.2.2 ICI + Chemotherapy

The NCT03513666 trial was a phase 2 study that evaluated toripalimab (anti-PD-1) + platinum doublet chemotherapy in patients with *EGFR*m advanced NSCLC who developed PD on first- and second-generation EGFR TKIs without T790M mutation (51). Median PFS was 7.0 months, and interestingly the authors identified that *TP53* co-mutation patients experienced significantly improved ORR compared to *TP53*wt patients (62% *vs* 14%, *p* = 0.04). This combination was found to have manageable safety profile and efficacy, and a follow up randomized Phase III trial (NCT03924050) that will compare this combination to standard chemotherapy with planned enrollment for 350 patients (65).

The CheckMate 722 trial (NCT02864251) is a currently active phase 3 study of patients with *EGFR*m, T790M-negative recurrent or stage IV NSCLC who have previously been treated with EGFR TKI therapy (66). Arm A comprises nivolumab + platinum-doublet therapy and Arm C involves platinum-doublet alone. Additionally, the KEYNOTE-789 trial (NCT03515837) is another currently active Phase III trial evaluating pemetrexed-platinum combined with pembrolizumab versus placebo in *EGFR*m advanced NSCLC that has progressed on EGFR TKI (67). The results of these studies will provide highly valuable information on the efficacy and safety of second line ICI + chemotherapy. The CheckMate 722 trial has the additional benefit of comparing this strategy to dual ICI therapy (Arms A versus B).

#### 2.2.3 ICI + EGFR TKI Therapy

A number of trials have evaluated combination ICI therapy and EGFR-targeted therapy in the second-line and beyond. These studies were predicated on pre-clinical studies that suggested added benefit to the combined approach in animal models (68–71). However, results have mostly been disappointing and in many cases demonstrated increased and severe toxicities. Nonetheless, several ongoing trials are assessing different combination therapies that will be of interest when results are available.

##### 2.2.3.1 Nivolumab + EGFR TKI

Arm E of the CheckMate012 study evaluated nivolumab + erlotinib in 21 *EGFR*m NSCLC patients (52). 20/21 patients had discontinued prior erlotinib treatment due to PD, and 1/21 patients was TKI-naïve. 3/21 patients had an OR to nivolumab + erlotinib, including the treatment naïve patient who also had atypical *EGFR* mutation status (double L858R, S768I). 24-week PFS was rate was 48%. The PFS for the previously TKI-tread patients (n=20) was 5.1 months. Overall, most patients had PD and were switched to other treatment regimens (52). Of note, the patients who responded were either PD-L1 positive (10% or 65%) or had unknown PD-L1 status. While not significantly efficacious in this small study, the dual nivolumab + erlotinib therapy was overall tolerated well, with no grade 4 or 5 TRAEs reported and 2/21 patients discontinuing study drugs due to toxicity.

##### 2.2.3.2 Durvalumab + EGFR TKI

The TATTON phase 1b study (NCT02143466) is highly significant in regard to the safety of combination EGFR-targeted + ICI combination therapy. In TATTON, osimertinib was combined with durvalumab in one of the three study arms to treat 23 patients with advanced *EGFR*m NSCLC that had progressed on previous EGFR TKI therapy (53). The ORR was 43%; however, significant safety concerns arose as 48% of patients had at least one grade 3 TRAE and 5/23 patients developed interstitial lung disease (ILD), leading all patients to discontinue the study. This study is very relevant now that osimertinib is the standard of care for first-line *EGFR*m NSCLC and as second-line for *EGFR*m NSCLC that progressed on a previous EGFR TKI.

In the CAURAL phase III study (NCT02454933), patients with *EGFR*m T790M-positive advanced NSCLC with PD after initial EGFR TKI therapy were randomized to receive either osimertinib or osimertinib + durvalumab (54). CAURAL was terminated early after one patient developed ILD given the contemporaneously reported results of the TATTON trial, though partial results were reported. In all, 15 patients received osimertinib and 14 received osimertinib + durvalumab. The ORR in the osimertinib arm was 80% versus 64% in the combination arm, and the median 12-month PFS rates were 82% and 76% for the osimertinib and combination arms respectively, indicating no evidence of increased efficacy of the combined approach. Aside from the one patient who developed grade 2 ILD in the combination arm (after receiving only a single dose of durvalumab and remaining on osimertinib), the safety profile was otherwise relatively unremarkable with no other ILD events reported.

##### 2.2.3.3 Atezolizumab + EGFR TKI

A phase 1b/2 study (NCT02630186) evaluating rociletinib, a third generation EGFR TKI, + atezolizumab in *EGFR*m patients who progressed after prior EGFR TKI was terminated after only three patients were recruited (72). No efficacy data were reported, and it was noted that 1/3 patients experienced a serious AE (pancreatitis), and all patients experienced AEs that included diarrhea (3/3), nausea (2/3), and bilateral hearing loss (1/3) among others.

##### 2.2.3.4 Tremelimumab + EGFR TKI

The phase 1 GEFTREM trial (NCT02040064) evaluated the safety of dose-escalation of the CTLA-4 inhibitor tremelimumab (3 mg/kg, 6 mg/kg, and 10 mg/kg) in combination with gefitinib in previously treated *EGFR*m NSCLC patients (73). The preliminary report indicated that dose-limiting toxicities occurred in 5/26 patients, and multiple grade 3/4 TREAs were reported that resolved upon discontinuation of tremelimumab. However, the overall safety profile of the 3 mg/kg tremelimumab + gefitinib combination was considered acceptable and an expansion cohort is planned.

##### 2.2.3.5 Ipilimumab + EGFR TKI

The phase 1 NCT01998126 study evaluated the addition of ipilimumab to erlotinib in *EGFR*m mNSCLC patients already on erlotinib for at least 28 days (74). Dose limiting toxicity (DLT) was reached in 3/8 patients, and excessive toxicity led to the study being closed after 14 patients. 4/11 *EGFR*m patients developed grade 3 colitis. However, PFS from start of ipilimumab was 17.9 months in 11 *EGFR*m patients, well above the typical observed for monotherapy, leading the authors to conclude that while ipilimumab + erlotinib caused excessive toxicity, targeted therapies with immunotherapy merited further study.

#### 2.2.4 Dual ICI Therapy

Cohort H of the KEYNOTE-021 phase 1/2 study assessed pembrolizumab 2 mg/kg plus ipilimumab 1 mg/kg as second-line or later therapy (55). Of the 10 *EGFR*m patients, only 1 (10%) patient responded to therapy compared to an ORR of 30% for the entire study population. 98% of patients experienced a TRAE, including 49% with grade 3-5 AEs.

Multiple active studies are investigating dual ICI therapy in second line or later *EGFR*m NSCLC. The ILLUMINATE Phase 2 study (NCT03994393) is evaluating the safety and tolerability of combined durvalumab and tremelimumab plus platinum-pemetrexed in *EGFR*m NSCLC following progression on EGFR TKIs (75). 100 patients will receive induction durvalumab + tremelimumab with platinum-pemetrexed every three weeks, followed by maintenance durvalumab + pemetrexed every four weeks until disease progression. *EGFR*m T790M negative and positive patients will be included. Additionally, two arms of the CheckMate 722 phase 3 trial, described above, will compare nivolumab + ipilimumab (Arm B) to platinum-doublet chemotherapy (Arm C). We eagerly await the results from these study that will leverage the potentially enhanced immune response of dual ICI therapy.

#### 2.2.5 ICI + VEGF inhibitor + Chemotherapy

The IMpower150 Phase 3 study (NCT02366143) assessed the addition of PD-L1 inhibition with atezolizumab and VEGF inhibition with bevacizumab to carboplatin + paclitaxel (CP) in patients with mNSCLC. Regimens included bevacizumab + CP (BCP), atezolizumab + CP (ACP), and atezolizumab + BCP (ABCP) (76). A subgroup analysis was performed of *EGFR*m patients; notably, 85-88% of the patients had previously received at least one EGFR TKI therapy (56). In the initial subgroup analysis, median OS was not reached with the ABCP group in *EGFR*m patients. Fortunately, the updated results were recently published and demonstrated that among *EGFR*m patients who had received prior TKI therapy a significant increase in median OS was observed with the ABCP regimen (27.8 months versus 14.9 months with ACP and 18.1 months with BCP) (77). The HR for ABCP versus BCP was 0.74. The toxicity profile was similar between different regimens in *EGFR*m patients: 64-68% of patients across all three regimens experienced at least one Grade 3-4 TRAE, and 1-3% experienced a Grade 5 TRAE. These results suggested the hopeful possibility of an effective ICI-combination therapy for patients who experienced PD on an EGFR TKI.

Lam et al. recently reported the results of the Phase II NCT03647956 trial that enrolled 40 patients with metastatic *EGFR*m NSCLC that had progressed on EGFR TKI (57.5% osimertinib) (57). Patients were treated with atezolizumab + bevacizumab + pemetrexed-carboplatin until progression. Median PFS was 9.43 months and median OS was not mature yet at time of publication (1-year OS was 72.5%). 37.5% of patients experienced a grade 3 or above TRAE but only 1/40 patients discontinued treatment due to toxicity. These encouraging results, coupled with the IMpower150 *EGFR*m subgroup results, support the potential efficacy of adding VEGF inhibition to ICI and chemotherapy as second-line in *EGFR*m NSCLC that has progressed on EGFR TKI.

The phase 2 NCT04517526 trial is planning to enroll 60 patients with stage IV *EGFR*m NSCLC with PD after first-line osimertinib. Patients will receive platinum-based chemotherapy + bevacizumab + durvalumab + stereotactic radiotherapy to oligometastatic or oligoprogressive sites (78). The results of this study will be of great interest as it will combine advanced combination immunotherapy and radiation therapy approaches.

## 3 Discussion

The treatment of *EGFR*m NSCLC has made significant progress with the advent of osimertinib, a third-generation TKI, that is now the standard of care for first-line treatment. Unfortunately, patients will almost uniformly experience PD. Meanwhile, the role of ICI therapy in *EGFR*m NSCLC is complex, with many studies describing additive toxicities without clinical benefit in combination ICI + EGFR TKI treatment models as described above. Given this, there is controversy around the standard of care for *EGFR*m NSCLC patients who have progressed on EGFR TKIs, with some advocating for chemotherapy alone and some advocating for chemotherapy combined with ICI therapy (79, 80). The National Comprehensive Cancer Network (NCCN) guidelines recommend tailoring response by symptomatology and location and number of metastatic sites, with osimertinib continuation recommended for asymptomatic *EGFR*m patients with PD on EGFR TKI and consideration of definitive local therapy for oligometastatic disease (81). For patients with symptomatic and widely metastatic PD on osimertinib, the NCCN guidelines recommend standard therapeutic strategies and clinical trial enrollment. The European Society of Molecular Oncology (ESMO) 2020 clinical practice guidelines recommend osimertinib as second-line if another EGFR TKI was utilized first-line and resistance is found to be due to the EGFR T790M mutation, followed by platinum doublet chemotherapy after progression on osimertinib (82). The ESMO guidelines briefly mention ICI therapy as a non-EMA approved option that can be considered after targeted therapies have been exhausted. Fortunately, active research is delineating important and unique characteristics in the tumor biology and TME of *EGFR*m NSCLC as well as identifying subgroups of *EGFR*m NSCLC patients who may have an improved response to ICI therapy, as discussed below.

### 3.1 Unique Biology of *EGFR*m NSCLC and Future Research Directions

**Figure 1:**
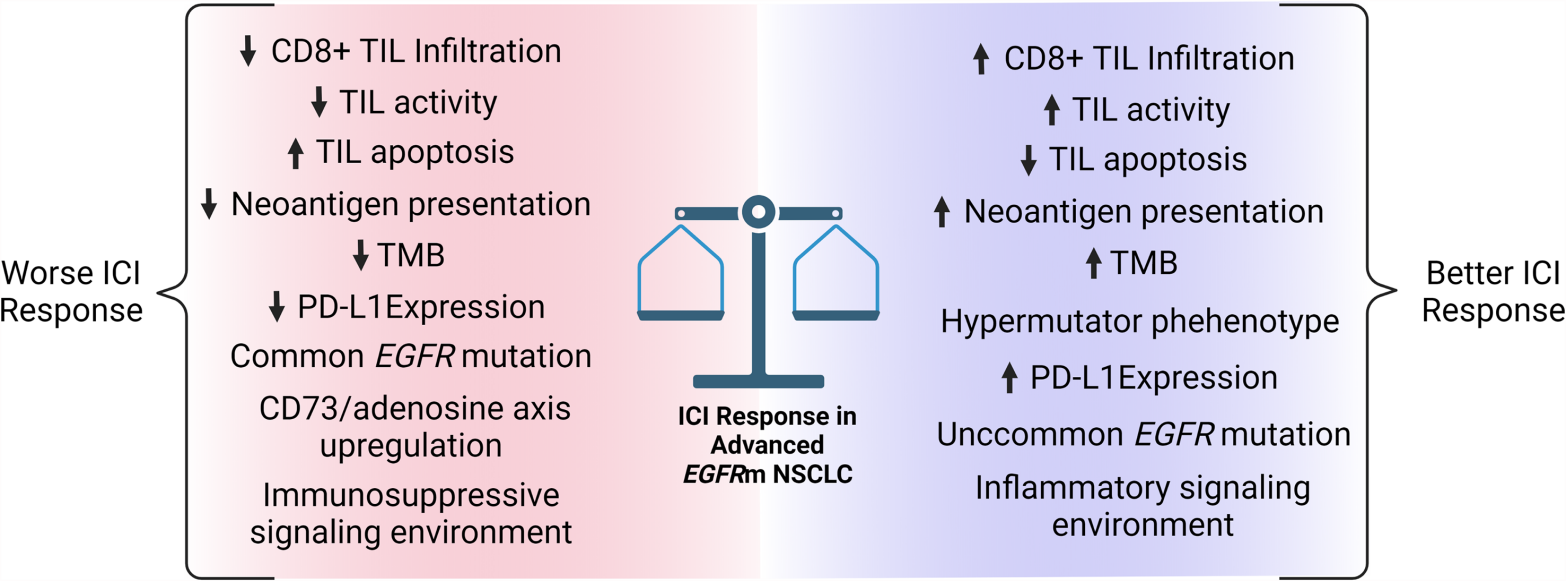
Factors that may influence immune checkpoint inhibitor response in advanced *EGFR*m NSCLC. ICI, immune checkpoint inhibitor; TIL, tumor infiltrating lymphocyte; TMB, tumor mutational burden; PD-L1, programmed cell death 1 ligand. Figure created with BioRender.com.

There is a growing appreciation that the TME is the master regulator of response to ICI therapy (83), and *EGFR*m NSCLC tumors are no exception with their unique and complex tumor biology. On average, *EGFR*m tumors generate an immunosuppressive TME with less PD-L1 expression, reduced TMB and neoantigen presentation, decreased TIL infiltration, and activation of the immunosuppressive CD73/adenosine axis, all of which decrease ICI efficacy (**Figure 1**). Furthermore, the standard biomarker for ICI therapy, PD-L1 TPS, has less straightforward utility in *EGFR*m tumors and biomarkers to predict ICI response are not yet standardized in *EGFR*m NSCLC patients. The unique aspects of *EGFR*m NSCLC tumor biology are active areas of research, with multiple areas of interest for current and future clinical trials (**Table 3**).

**Table 3:** Active or planned clinical trials addressing important questions of ICI use in advanced *EGFR*m NSCLC.

Trial	Phase		Population	n	Intervention	Primary End Point(s)	Status
**Question: Activity of second-line dual ICI therapy**							
ILLUMINATE/NCT03994393	2	*EGFR*m NSCLC that failed third generation TKI		100	Durvalumab + Tremelimumab + Platinum-Pemetrexed	OTRR*	Recruiting
CheckMate722/NCT02864251	3	*EGFR*m NSCLC that failed first- or second-line EGFR TKI therapy		365	(Arm B) Nivolumab + Ipilimumab *vs* (Arm C) Platinum-doublet	PFS	Active
**Question: Activity of second-line combination ICI + chemotherapy**							
NCT03924050	3	Advanced *EGFR*m NSCLC that has progressed on EGFR TKI		350	Toripalimab + standard chemotherapy	PFS	Recruiting
CheckMate722/NCT02864251	3	*EGFR*m NSCLC that failed first- or second-line EGFR TKI therapy		365	(Arm A) Nivolumab + Platinum-doublet *vs* (Arm C) Platinum-doublet	PFS	Active
KEYNOTE-789/NCT03515837	3	EGFR*m* NSCLC resistant to EGFR TKI		492	Pembrolizumab + Pemetrexed + Chemotherapy *vs* Placebo + Pemetrexed + Chemotherapy	PFS, OS	Active
**Question: Activity of second-line ICI + chemotherapy + antiangiogenic therapy**							
NCT04517526	2	Stage IV *EGFR*m NSCLC that has progressed on EGFR-TKI		60	Pemetrexed + Cisplatin/Carboplatin + Bevacizumab + Durvalumab + SBRT	PFS, OS	Not yet recruiting
**Question: Activity of CD73/adenosine axis inhibition + ICI therapy in EGFRm NSCLC**							
No active studies*							
**Question: Activity of TNF-α agents + ICI therapy in EGFRm NSCLC**							
*No active studies*							

Trial information obtained from ClinicalTrials.gov *A phase 1b/2 trial of oleclumab (CD73-ab) + osimertinib versus AZD4635 is currently recruiting (NCT03381274). This study does not have an ICI arm, but will provide helpful information on the utility and tolerability of oleclumab in EGFR NSCLC patients. OTRR, overall treatment response rate; PFS, progression-free survival; OS, overall survival; SBRT, stereotactic body radiation therapy.

#### 3.1.1 PD-L1 Expression

PD-L1 TPS is a standard biomarker for stratifying patients in clinical trials of ICI therapy in *EGFR*wt cells, with evidence from multiple trials that higher PD-L1 TPS tumors (e.g. with TPS ≥ 50%) have an enhanced response to ICI therapy (84, 85). Of note, PD-L1 expression is not uniformly prognostic of response to ICI therapy (86), and it has been demonstrated that NSCLC patients without PD-L1 immunohistochemical staining can still derive benefit from ICI therapy (87), supporting the now widely accepted notion that PD-L1 status by itself is insufficient to predict ICI response.

In *EGFR*m NSCLC, the value of PD-L1 expression is even less clear. Mechanistic studies have demonstrated that upregulation of *EGFR* signaling *in vitro* leads to increased PD-L1 expression by pathways including the IL-6/JAK/STAT3 pathway (88). However, immunohistochemical and mRNA expression profiling analysis of *EGFR*m NSCLC patient tumor samples demonstrated decreased PD-L1 expression across multiple datasets (89, 90), leading to an unresolved discrepancy between preclinical and clinical studies. Of note, a weakness of these studies is the inability to assess the half-life of PD-L1 between subgroups.

In regard to treatment response, multiple studies have demonstrated that increased PD-L1 expression on *EGFR*m NSCLC cells predicts worse outcomes with TKIs but improved outcome with ICI therapy (91–93). Liu et al. recently published a correlation analysis of 57 *EGFR*m NSCLC patients who received ICI treatment after developing PD on EGFR TKIs (94).They identified by using a TKI-PFS cutoff of 10 months that *EGFR*m patients with <10 month TKI-PFS had significantly improved ICI-PFS of 15.1 versus 3.8 months, respectively (HR 0.26, 95% CI: 0.12-0.5, *p* = 0.0002), strongly suggesting that *EGFR*m tumors are either TKI or ICI responsive. Intriguingly, this relationship was independent of PD-L1 status, again reiterating the importance of other elements of the TME in treatment response. To further probe differences in the TME between these groups, they performed single-cell RNA sequencing of patients with TKI-PFS <10 months (group A) and >10 months (group B). Group A demonstrated significantly higher proportion of T-cell TILs along with increased CD8+ effector proportion of T-cells. These results suggest a critical role for CD8+ effector TILs in determining response to ICI therapy in *EGFR*m NSCLC, as discussed in more detail below.

While PD-L1 expression is not uniformly predictive of ICI response, there is some evidence that *EGFR*m NSCLC tumors with increased PD-L1 TPS have improved response to third or later line ICI therapy (63). Thus, while ICI monotherapy is inappropriate for first line treatment as described above, PD-L1 analysis may be a valuable component of a holistic evaluation of the TME in *EGFR*m, along with other elements including TMB, TILs, and other discussed below to assist oncologists in deciding on later-line ICI treatment strategies for *EGFR*m patients who fail EGFR TKI therapy. As such, future clinical trials should continue to gather and report PD-L1 expression data so that these relationships can be better elucidated.

#### 3.1.2 Tumor Infiltrating Lymphocytes

The efficacy of ICI therapy depends on the intratumoral migration and activation of CD8^+^ effector T-cells where they perform cytotoxic functions after interaction of the T-cell receptor with tumor-specific peptides displayed on MHC-I complexes on tumor cells (29). Multiple studies have demonstrated that *EGFR*m NSCLC tumors have reduced CD8^+^ TIL presence compared to *EGFR*wt tumors (89, 95, 96). Interestingly, Zhao et al. recently published an analysis of 190 surgical lung ADC samples that demonstrated increased apoptosis in the *EGFR*m patient tumor samples (96). They further went on to demonstrate that exosomes secreted from *EGFR*m cells were more capable of inducing CD8^+^ T-cell apoptosis *in vitro* than exosomes from *EGFR*wt cells. These results suggest that, in addition to reduced TIL density in *EGFR*m tumors, there may also be increased TIL apoptosis that impairs immune-mediated tumor destruction. Further study of this mechanism may provide valuable new information on the TME in *EGFR*m NSCLC and possibly provide a novel therapeutic avenue to enhance antitumor immunotherapy (97).

Strategies to increase TIL trafficking and activity in tumors including NSCLC are an active area of research (98). Possible approaches include targeted tumor necrosis factor α (TNF-α) delivery and anti-angiogenic drugs including inhibitors of VEGF. TNF-α causes endothelial cell activation and increased vessel permeability that can enhance the ability of both chemotherapy and immune cells to penetrate solid tumors (99); however, systemic TNF-α administration is quite toxic (100). A compound containing the tumor vasculature-homing peptide Cys-Asn-Gly-Arg-Cys (NGR) has been fused to TNFα to create a tumor vasculature-homing version of TNF-α that avoids the toxicity of systemic TNFα administration (NGR-hTNF) (101). A phase II trial of NGR-hTNF combined with chemotherapy in patients with chemotherapy-naïve NSCLC was previously reported. Patients with nonsquamous NSCLC in the chemotherapy + NGR-hTNF arm experienced improved PFS at 8 months (38% versus 18% with chemotherapy alone) and a tolerable safety profile (102). While preclinical models support the ability of TNF-α to also enhance ICI therapy (103), there are no active clinical trials involving NGR-hTNF and ICI therapy in NSCLC due to the manufacturer of NGR-hTNF discontinuing the product after a Phase III mesothelioma trial did not meet its primary endpoint (104). Importantly, an NGR-TNF derivative with an additional serine at the N-terminus that demonstrates increased stability, S-NGR-TNF, has been recently developed (105). It will be intriguing to see if TNF-α strategies such as S-NGR-TNF can restore TIL trafficking, enhance ICI therapy, and augment chemotherapy delivery to *EGFR*m NSCLC tumors.

VEGF is also known to suppress TILs *via* multiple mechanisms, including suppressing endothelial cell activation, inhibiting TNFα-mediated gene regulation, and blocking dendritic cell maturation thereby reducing T-cell activation (106, 107). In preclinical models, VEGF inhibition synergized with PD-1 blockade and reduced T-cell exhaustion, and in clinical studies combinations of therapies including ICIs and TKIs have demonstrated improved TIL recruitment and improved PFS (108). The IMpower150 study, for example, demonstrated improved PFS of *EGFR*m NSCLC patients who had disease progression on or did not tolerate an EGFR TKI when they were treated with a combination of chemotherapy, ICI therapy, and VEGF-inhibition as described above (56, 76). The success of VEGF-inhibition in second-line combination chemotherapy + ICI therapy for *EGFR*m NSCLC patients with PD on EGFR TKI is one of the few bright signals currently in the field, and we eagerly await the results of current trials that are ongoing further exploring this question (**Table 3**).

#### 3.1.3 Tumor Mutation Burden

On average, *EGFR*m NSCLC patients have a decreased TMB compared to *EGFR*wt patients (89, 109). This is thought to be at least partly due to the fact that *EGFR*m patients tend to have a lighter smoking history. Increased TMB classically correlates to decreased response to chemotherapy and an increased response to ICI therapy in NSCLC (110, 111), and in *EGFR*m NSCLC patients increased TMB correlates negatively with response to EGFR TKIs (112). Increased TMB is thought to potentiate ICI therapy by creating an environment where more tumor-specific neoantigens are generated, thus creating more targets for TILs to recognize and enhancing the adaptive anti-tumoral response. Indeed, significantly fewer candidate MHC class-I neoantigens were identified in *EGFR*m versus *EGFR*wt NSCLC tumors in a whole-genome DNA sequencing study (113). While the decreased TMB in *EGFR*m NSCLC patients overall may contribute to decreased efficacy of ICI therapy, there is some evidence that TMB may still be of significance in this population. For example, Hastings et al. retrospectively analyzed 171 cases of *EGFR*m NSCLC and demonstrated that *EGFR^Δ19^* tumors had a lower TMB and a worse response to ICI therapy compared to *EGFR* ^L858R^ tumors (114). Additionally, certain hypermutator phenotypes such as DNA mismatch repair (MMR) deficient tumors and DNA polymerase delta and epsilon proofreading mutants, while uncommon in NSCLC, may respond well to ICIs (115, 116). As such, subgroups of *EGFR*m patients with increased TMB, while less common than in *EGFR*wt context, are predicted to still receive increased benefit from ICI therapy compared to their low TMB counterparts. TMB analysis is an intriguing and significant element that should be strongly considered for clinical studies of *EGFR*m NSCLC patients.

#### 3.1.4 CD73/Adenosine Axis

CD73 is an ecto-nucleotidase that catabolizes the breakdown of extracellular ATP to adenosine (117). There is a growing appreciation that the CD73/adenosine axis plays a significant and complex role in the TME. Increased intratumoral adenosine contributes to localized immunosuppression and impairment of T-cell effector function (118, 119), and the CD73/adenosine axis is becoming considered an immune checkpoint in its own right (120). Pre-clinical data demonstrated that anti-CD73 monoclonal antibodies (mAbs) significantly enhanced the activity of anti-CTLA-4 and anti-PD-1 mAbs in animal studies of colon, prostate, and breast cancer (118). An intriguing, recently published study by Le et al. analyzed upregulated genes in *EGFR*m NSCLC tumors and found that two of the top upregulated genes (*NT5E* and *ADORA1*) belonged to the CD73/adenosine pathway (89), suggesting that *EGFR*m NSCLC may leverage the CD73/adenosine axis to generate an immunosuppressive TME. They assessed the efficacy of an anti-CD73 mAb in a mouse model of *EGFR*m murine lung cancer and found that anti-CD73 treatment significantly reduced tumor size. As such, an active question is whether suppression of the CD73/adenosine axis can enhance the treatment of *EGFR*m NSCLC. Along these lines, a human mAb targeting CD73, Oleclumab, is being assessed in a phase 1b/2 study (NCT03381274) in combination with either osimertinib or AZD4635, which is an adenose 2a receptor (A2aR) inhibitor (121). Given the encouraging preclinical data, we eagerly look forward to further clinical trials utilizing anti-CD73 mABs or A2aR inhibitors in conjunction with ICI therapy.

#### 3.1.5 Role of Specific EGFR Mutations

There is intriguing evidence that the specific *EGFR* mutation impacts the immunogenicity of the TME and response to ICI therapy. Chen et al. performed a large single-study of 600 NSCLC patients in China with *EGFR*m NSCLC and identified 49 with uncommon mutations (Ex20ins, S767I, L861Q, G719X, and double mutations) (91). They found a much higher proportion of PD-L1 expressing tumors with uncommon mutations compared to classic mutations (49% versus 12.2%), and CD8^+^ TIL infiltration was more abundant in this group (91). They reported worse OS for patients with PD-L1 positive *EGFR*m NSCLC versus PD-L1 negative (median OS 15.2 versus 29.3 months, *p* = 0.006), though most of these patients received EGFR TKI monotherapy across all lines of treatment. Negrao et al. reported that metastatic *EGFR*m exon 20 mutation NSCLC patients had increased benefit from ICIs compared to classic mutation patients (exon 19 del, exon 21 L858R) with an ORR of 25% versus 0% and disease control rate (DCR) of 50% versus 15% (122). Mazieres et al. analyzed the IMMUNOTARGET registry and compared the molecular characteristics of *EGFR*m patients to response to ICIs (123). *EGFR* exon21 mutation patients derived significantly longer PFS from single-agent ICI therapy (2.5 months) in this database than patients with T790M and exon 19 mutations (1.4 and 1.8 months, respectively, *p <*0.001). As such, these studies suggest that uncommon *EGFR* mutations including exon 21 mutations may have increased immunogenicity and response to ICIs. Future clinical trials should ensure that the specific *EGFR* genetic alterations are reported and provide mutation subgroup data so that further evidence on this subject can be obtained.

#### 3.1.6 ICI Response Prediction

One of the greatest needs in the field currently is the development of a scoring/stratification system that will predict which *EGFR*m patients will benefit from ICI therapy. As more studies publish the results of detailed molecular and immunohistochemical analysis, this will empower a more comprehensive understanding of the cellular composition of *EGFR*m TMEs and tumor biologies (**Figure 1**). Complex multivariate analyses should be employed to delineate subgroups of *EGFR*m patients that will benefit from ICI therapy. The use of artificial intelligence (AI) including artificial neural networking is being studied for the analysis of TMEs (124, 125), and may prove invaluable to identify signatures of *EGFR*m tumors that predict ICI response. The value of machine learning in *EGFR*m tumor biology was recently demonstrated by Song et al. who utilized a machine learning model to analyze pre-treatment computed tomography (CT) images of stage IV *EGFR*m NSCLC patients (126). Their machine learning approach successfully identified an imaging signature able to stratify *EGFR*m patients most likely to rapidly progress despite TKI therapy. It is a logical next step to apply machine learning to stratify patients likely to respond to ICI therapy based on tumor biological characteristics.

#### 3.1.7 Effect of EGFR TKIs on the TME

Multiple lines of pre-clinical evidence suggested synergy between EGFR TKI inhibition and ICI therapy. In pre-clinical studies, EGFR inhibition enhanced antigen presentation to T-cells, stimulated immunogenic apoptosis of tumor cells, boosted T-cell chemoattractants, and stimulated MHC-1 upregulation, all of which are predicted to enhance the anti-tumor immune response (68–71). Despite this, early clinical studies demonstrated that preclinical studies would not translate in a straightforward manner. IHC analysis of tumors from early *EGFR*m patients treated with ICIs, against expectation, demonstrated decreased PD-L1 expression and decreased CD8+ TILs (95), data that has since been recapitulated in multiple studies described above. This has led to the active research question of the effect of EGFR TKIs on the TME of *EGFR*m NSCLC *in vivo* during and after therapy.

Multiple groups have addressed this question with TME analysis at various time points of treatment. Isomoto et al. performed serial immunohistochemical analysis of 138 patients who underwent rebiopsy after progression on EGFR TKI treatment (127). They found multiple significant changes in the TME after PD, including an expanded proportion of high (≥50%) PD-L1 expressing tumors and decreased CD8+ TILs in PD-L1 <50% tumors. Notably, they identified subgroups with opposing clinical courses: tumors with high PD-L1 expression after progressing on EGFR TKI had significantly longer PFS with ICI therapy (7.1 versus 1.7 months, *p* = 0.0033) and increased CD8^+^ TIL presence. In contrast, PD-L1 <50% tumors had significantly decreased CD8^+^ TIL density. Also of interest, the PD-L1 high tumors had increased FOXP3+ and CD73 TIL density, suggesting that regulatory T-cell (Treg) and CD73 axis activation may contribute to ICI treatment failure.

Sugiyama et al. analyzed surgically resected *EGFR*m tumors and found decreased CD8+ TILs and increased FOXP3+CD4+ Tregs, further supporting a role for Treg suppression of the immune response in *EGFR*m tumors (128). Gurule et al. performed RNA sequencing of patient tumors before and 2 weeks after TKI treatment and demonstrated induction of an interferon response program (71). Interestingly, higher enrichment of interferon gamma (IFNγ) was correlated with longer time to progression. Taken together, these results suggest that *EGFR*m NSCLC tumors undergo diverse responses to EGFR TKIs with some tumors becoming more immunogenic and some becoming more immunosuppressive with resulting divergent responses to ICI therapy (129). While the mechanism behind the divergent TME responses to TKI therapy in *EGFR*m NSCLC is unclear, these studies suggest that rebiopsy may have clinical benefit in identifying subpopulations of patients who are more likely to respond to ICI as subsequent therapy.

### 3.2 ICI + EGFR TKI Toxicity

Despite the pre-clinical evidence of synergy between EGFR TKIs and ICI therapy, the clinical trials of combined or sequential ICI and EGFR TKI therapies as described above failed to demonstrate additive clinical benefit and generated safety concerns in two major regards. First, multiple combination of ICI + EGFR TKI therapies were found to generate severe toxicities. As described above, durvalumab + gefitinib and pembrolizumab + gefitinib were correlated with high grade hepatotoxicity (40, 41), durvalumab + osimertinib was correlated with an increased incidence of ILD (53), and both azetolizumab + erlotinib and ipilimumab + erlotinib were poorly tolerated with an increased risk of various grade 3/4 TRAEs (42, 74). In line with these studies, Oshima et al. performed an analysis of adverse events reported through the FDA adverse event reporting system and compared the incidence of interstitial pneumonitis (IP) between patients treated with and EGFR TKI, nivolumab, or combination nivolumab + EGFR TKI (130). Their analysis identified a significant elevation in IP in the combination group (25.7%) versus 6.4% for nivolumab alone and 4.6% for EGR TKI alone, suggesting an additive interaction between EGFR TKIs and nivolumab in favor of developing IP. We note that certain ICI + EGFR combinations were well tolerated, including pembrolizumab + erlotinib and nivolumab + erlotinib (41, 52). Given the small number of patients in most of these studies, caution must be taken in interpreting these results, though a clear theme of concerning safety signals without added benefit for most tested ICI + EGFR TKI strategies emerges.

Second, the sequence of ICI and TKI therapies appears critical in determining toxicity. Schoenfeld et al. analyzed 126 patients treated with ICI and EGFR TKI at a single institution in various sequences and found that 15% of patients treated with ICI followed by osimertinib developed severe irAEs whereas 0% of patients treated with osimertinib followed by ICI therapy developed severe irAEs (131). In other words, osimertinib after ICI was dangerous, whereas ICI after osimertinib was tolerated. This study is congruent with findings reported above, including the increased incidence of TRAEs in patients who experienced PD on first-line ICI monotherapy and then switched to second-line EGFR TKI in the KEYNOTE-001 study (38, 58), as well as the lack of any increased toxicity noted in patients who switched to either pembrolizumab, nivolumab, or atezolizumab after PD on first-line EGFR TKI therapy (44–46). Given these combined results, we urge oncologists not to empirically start advanced NSCLC patients on ICI therapy until the oncogene status of their cancer is known, as inadvertent ICI treatment of *EGFR*m NSCLC will increase the risk of severe TRAEs on subsequent EGFR TKI therapy.

While the mechanism for checkpoint inhibitor toxicity is currently unknown, Zhai et al. recently reviewed possible causes that may include increased immune activity against cross-antigens in tumor and normal tissues, increased levels of pre-existing autoantibodies, and increased inflammatory cytokines in patients who experience irAEs (132). Given that EGFR inhibitors have been demonstrated to increase the expression of MHC class I and class II molecules (133), this suggests that increased autoreactivity stimulated by increased expression of cross-antigens *via* MHC class I and II molecules may at least a partially explain severe TRAEs such as IP. One possible approach to combine ICI and EGFR TKI therapy more safely would be to target treatments specifically to tumor cells, for example by tumor-homing nanoparticles (134), thereby bypassing adverse effects due to systemic impact of the drugs. Alternatively, if biomarkers predicting which patients are at risk of developing severe TRAEs from combination ICI + EGFR TKI can be identified, then patients could be stratified by likelihood to develop severe combination TRAEs so that combination therapy could be applied more safely in a first line setting. In this way, future clinical trials could attempt to realize the potential of combination ICI + EGFR TKI therapy seen in preclinical studies.

## 4 Concluding Remarks

NSCLC remains the deadliest malignancy on the planet. 15-67% of NSCLC tumors harbor *EGFR* mutations based on geographic region, lending urgency to the development of better therapeutic strategies for *EGFR*m NSCLC patients. First-line ICI therapy is clearly inferior to *EGFR*-targeted therapy, and first-line combination EGFR TKI + ICI therapy has so far demonstrated synergy only in regard to toxicity without any consistent clinical benefit. Furthermore, pre-treatment of *EGFR*m NSCLC patients with ICIs can prime patients for serious TRAEs on subsequent EGFR TKI therapy due to an unknown mechanism. As such, oncologists must take great caution to avoid treating NSCLC patients with ICI therapy until molecular analysis has been performed and *EGFR* mutation status is ascertained.

While first-line ICI monotherapy and ICI + EGFR TKI combination therapy in *EGFR*m NSCLC patients has thus far been disappointing, intriguing results have been obtained from trials of second-line ICI therapy combinations and multiple open research questions are under clinical investigation (**Table 3**). Recent trials have demonstrated encouraging signals with dual ICI blockade and combination of ICI, chemotherapy, and VEGF inhibitors. Furthermore, accumulating evidence suggests that multiple components of *EGFR*m tumor biology may predict response to ICI therapy, including specific *EGFR* mutation, TMB, PD-L1 expression, and TIL density among others (**Figure 1**). Multiple active areas of research are identifying other significant *EGFR*m TME differences including CD73/adenosine axis activation that may prove fruitful for the development of novel therapeutic interventions to enhance the immunogenicity of *EGFR*m tumors (135). As such, there exists a great deal of hope for improved therapies for *EGFR*m NSCLC patients in the near future. We believe that as researchers and clinicians continue to advance our understanding of *EGFR*m NSCLC tumor and TME biology that outcomes for patients will only continue to improve.

## Author Contributions

NW wrote the manuscript. RM provided major editorial and intellectual contributions. UM, KS, YL, and YZ provided significant intellectual contributions. All authors contributed to the article and approved the submitted version.

## Funding

Departmental funding (RM) was utilized for publishing costs.

## Conflict of Interest

RM has participated in advisory boards for AstraZeneca, Guardant Health, Novocure, and Takeda, and consulting for AstraZeneca. YL has participated in advisory boards for AstraZeneca and Novocure.

The remaining authors declare that the research was conducted in the absence of any commercial or financial relationships that could be construed as a potential conflict of interest.

## Publisher’s Note

All claims expressed in this article are solely those of the authors and do not necessarily represent those of their affiliated organizations, or those of the publisher, the editors and the reviewers. Any product that may be evaluated in this article, or claim that may be made by its manufacturer, is not guaranteed or endorsed by the publisher.
